# Pyogenic liver abscess and sepsis caused by mixed anaerobic bacteria in an immunocompetent adult: a case report

**DOI:** 10.3389/fmed.2025.1486256

**Published:** 2025-02-13

**Authors:** Ziwen Fan, Mu Mei, Cen Chen

**Affiliations:** Department of Respiratory Medicine, Changde Hospital, Xiangya School of Medicine, Central South University (The First people’s Hospital of Changde City), Changde, China

**Keywords:** liver abscess, sepsis, anaerobic bacterial, metagenomic next-generation sequencing (mNGS), immunocompetent adult infection

## Abstract

**Background:**

Anaerobic bacterial communities in the digestive tract play an important role in digestive tract infections and aspiration pneumonia. However, ectopic infections originating from these communities are uncommon.

**Case report:**

We present a rare case of a 64-year-old immunocompetent female was admitted with no significant medical history who developed a pyogenic liver abscess and sepsis caused by multiple anaerobic bacteria of digestive tract origin. Metagenomic next-generation sequencing (mNGS) detected four types of anaerobic bacteria in both peripheral blood and abscess puncture fluid. Culture confirmed the presence of three of these microorganisms. Treatment with a combination of meropenem and metronidazole resulted in the patient’s subsequent recovery and discharge.

**Conclusion:**

This report highlights the occurrence of ectopic infections caused by multiple anaerobic bacteria leading to pyogenic liver abscess and sepsis, underscoring the importance of considering anaerobic bacteria and conducting rapid comprehensive pathogen detection in clinical practice.

## 1 Introduction

Pyogenic liver abscess (PLA), also referred to as bacterial liver abscess, is a localized purulent liver lesion caused by bacterial invasion (1). The incidence of PLA has been steadily rising in recent years, causing concerns among healthcare professionals. PLA mainly affects individuals with compromised immune systems, and without prompt detection and treatment, it poses a significant risk of mortality (2). In the Asian region, Klebsiella pneumoniae is the predominant microorganism responsible for PLA, while occurrences of PLA and sepsis due to digestive tract anaerobic bacterial infections are infrequent (3).

Herein, we present an immunocompetent adult infected with four types of digestive tract anaerobic bacteria leading to pyogenic liver abscess (PLA) and sepsis within a short timeframe. To the best of our knowledge, this is the first documented case of PLA and sepsis resulting from a mixed infection of digestive tract anaerobic bacteria in an immunocompetent woman.

## 2 Case presentation

A 64-year-old woman presented to our emergency department with a complaint of fatigue lasting over 2 weeks, fever persisting for 10 days, and 3 days of dyspnea. Her past medical history included chronic superficial gastritis, chronic colitis, and a previous rectal polyp removal more than a year ago. The patient underwent regular biannual health check-ups and had no known history of diabetes mellitus, malignancies, autoimmune disorders, immunocompromised conditions, or obesity. There was no history of smoking, alcohol abuse, or consumption of unhygienic food, and no positive family history of medical issues was reported.

**FIGURE 1:**
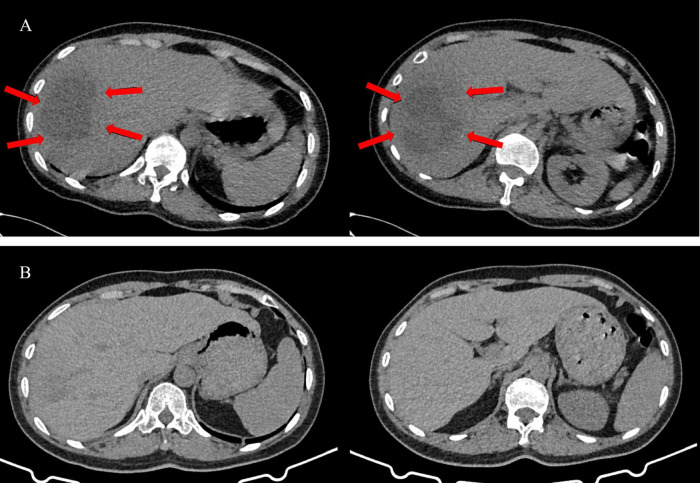
The abdominal CT images of the patient before and after treatment. **(A)** The abdominal CT images before treatment. Multiple round-shaped low-density lesions in the right lobe of the liver (Red arrows), with the largest lesion measuring approximately 75 × 56 mm. **(B)** The abdominal CT images after treatment. The low-density lesions appears were significantly reduced.

Upon arrival at the emergency department, her physical examination revealed a body temperature of 38.8°C, a heart rate of 127 beats per minute, and a blood pressure of 96/50 mmHg. Lung auscultation revealed slightly coarse breath sounds. Comprehensive examinations of the abdomen, nervous system, teeth, and oral mucosa showed no abnormalities. A comprehensive set of laboratory tests was conducted, yielding the following results: a lactate level of 2.5 mmol/L, an oxygenation index of 140, a white blood cell count of 13.6 × 10^9/L, a neutrophil count of 13.0 × 10^9/L, a hemoglobin level of 103 g/L, an alanine aminotransferase (ALT) level of 88 U/L, an aspartate aminotransferase (AST) level of 119 U/L, a serum albumin level of 25 g/L, an NT-proBNP level of 15,535.8 pg/ml, and a procalcitonin level of 33 ng/mL. The SARS-CoV-2 nucleic acid test returned negative. Additionally, serological tests for human immunodeficiency virus (HIV), hepatitis B virus, and hepatitis C virus were all negative. Chest CT scan revealed no significant abnormality. However, multiple round-shaped low-density lesions were detected in the right lobe of the liver, with the largest lesion measuring approximately 75 × 56 mm (Figure 1). The emergency department physician promptly initiated empiric treatment with imipenem-cilastatin as an antimicrobial therapy. However, the patient’s blood pressure dropped to 77/50 mmHg after 1 h, indicating the onset of septic shock. Subsequently, We promptly initiated rapid intravenous fluid resuscitation and administered norepinephrine to stabilize the blood pressure within the normal range. Within the next 12 h, arterial blood gas analysis revealed a progressive decrease in the oxygenation index, prompting the transfer of the patient to the Respiratory Intensive Care Unit (RICU) for continued treatment.

**FIGURE 2:**
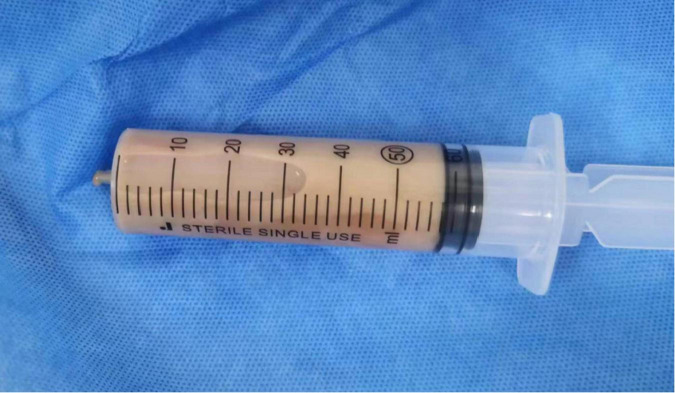
Brown malodorous liver abscess fluid obtained from the patient through ultrasound-guided needle aspiration procedure.

**FIGURE 3:**
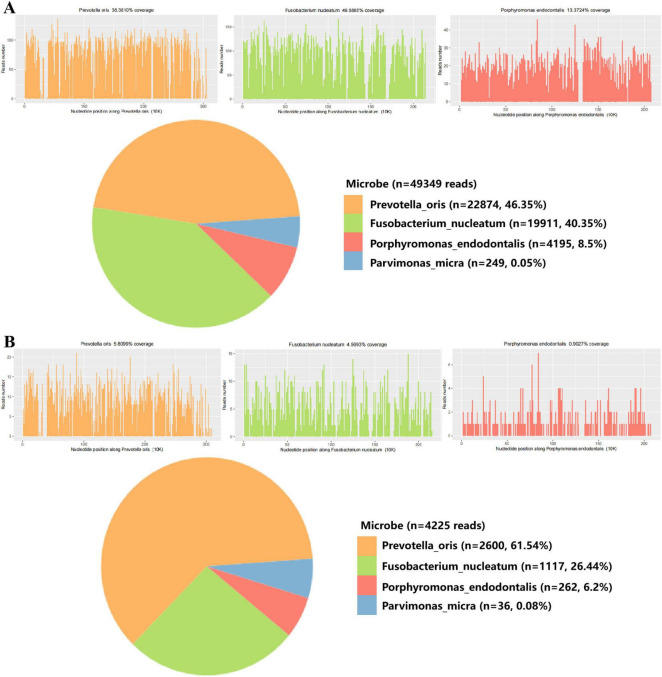
mNGS analysis of blood and liver abscess fluid from the patient. **(A)** mNGS results of liver abscess fluid sample. **(B)** mNGS results of liver abscess blood sample.

Upon transfer to the RICU, a follow-up examination revealed a white blood cell count of 33.1 × 10^9/L, a neutrophil count of 30.2 × 10^9/L, a procalcitonin level exceeding 100 ng/ml, a C-reactive protein value is 178 mg/L, and a serum IL-6 of 9980 pg/ml. Abdominal ultrasound confirmed the presence of multiple mixed nodules within the liver, with the largest one measuring 71 × 69 mm and situated in the posterior segment of the right lobe. Infection with Klebsiella pneumoniae was suspected, imipenem-cilastatin was discontinued and empirical administration of meropenem (1 g q6h) was initiated. An ultrasound-guided needle aspiration procedure was performed, draining brown malodorous liver abscess fluid (Figure 2) for culture and metagenomic next-generation sequencing (mNGS) analysis to identify causative pathogens. The mNGS results from both blood and liver abscess fluid confirmed the presence of four types of anaerobic bacteria of digestive tract origin, including *Prevotella oris*, *Fusobacterium nucleatum*, *Porphyromonas endodontalis*, and *Parvimonas micra* (Figure 3). Upon receipt of these results, the treatment plan was promptly adjusted to include a combination of meropenem (1 g q6h) and metronidazole (0.5 g qd) therapy. Daily irrigation of the liver abscess with metronidazole was also initiated. Despite a thorough reexamination of the patient’s oral cavity revealing no abnormalities, subsequent blood cultures yielded positive results for both *Prevotella oris* and *Parvimonas micra*, and the culture of liver abscess fluid confirmed the presence of *Fusobacterium nucleatum*. Besides, the patient underwent a comprehensive battery of tests and auxiliary examinations during hospitalization, all yielding normal results.

After 8 days of comprehensive treatment, the patient’s symptoms significantly improved. Peripheral blood leukocyte count decreased to 16 × 10^9/L, neutrophil count to 13 × 10^9/L, and serum IL-6 levels to 14 pg/mL. A subsequent abdominal ultrasound showed a marked reduction in the size of the mass, leading to the removal of the drainage tube. The patient continued using meropenem and metronidazole for 14 days. Observing further symptomatic improvement, radiological resolution, and substantial decline in infection-related laboratory markers, the antimicrobial regimen was de-escalated to cefoperazone/sulbactam and levofloxacin for an additional 14 days, until all clinical symptoms resolved completely. Two months later, a follow-up abdominal ultrasound confirmed the resolution of the liver abscess.

## 3 Discussion

This report presents a rare case of a 64-year-old immunocompetent woman who developed a liver abscess and sepsis caused by the mixed infection of digestive tract anaerobic bacteria. Early and rapid identification of causative agents is the key step for the successful treatment and management of this patient.

PLA accounts for approximately 80% of all cases of liver abscess (4). While the incidence of PLA varies regionally, it has been on the rise globally in recent years (5–7). Typical risk factors for liver abscess comprise diabetes mellitus, immunosuppression, intra-abdominal malignancies, a history of liver transplantation or biliary surgery. Typical presentations of PLA encompass fever and right upper abdominal pain. Common laboratory abnormalities include low serum albumin levels, elevated hepatic enzymes, and increased white blood cell count. An elevated serum procalcitonin level is particularly notable in PLA caused by bacteria (8–10). Similar to most PLA cases, the clinical symptoms, signs, and auxiliary examination results of our patient do not show any specificity. This significantly increases the difficulty of etiological diagnosis.

Regional variations exist in the microorganisms associated with PLA between Asia and Europe/America. In Asia, Klebsiella species, particularly Klebsiella pneumoniae, are the most prevalent pathogens, responsible for the majority of identified cases. Additionally, common genera consist of Escherichia coli, Enterococcus, Streptococcus, among others. 93.0% of cases in Asia are attributed to single bacterial infections (11, 12). In Europe and America, multiple bacterial infections are more common, with a higher proportion of liver abscesses caused by Escherichia coli and Streptococcus species. In contrast, liver abscesses caused by anaerobic bacteria are less common (4, 13–15). Researchers believe that the positivity rate for anaerobic bacteria may be underestimated due to the difficulty of culture. In this case, the patient’s blood and liver abscess fluid were tested using mNGS, with *Prevotella oris*, *Fusobacterium nucleatum*, *Porphyromonas endodontalis*, and *Parvimonas micra* detected. Additionally, *Prevotella oris* and *Parvimonas micra* were positive by blood culture, while *Fusobacterium nucleatum* positive by abscess fluid culture. Currently, there is no reported cases of liver abscess and severe bacteremia solely caused by mixed digestive tract anaerobic bacteria. The four types of bacteria identified in this case are all anaerobes that can be found in the normal oral and gastrointestinal microbiota. They are commonly detected in infections in the oral region such as periodontitis and periapical infections (16). In previous literatures, only one case of liver abscess caused by *Prevotella oris* in a patient with a history of diabetes has been reported (17). Liver abscesses caused by *Fusobacterium nucleatum* are more common than those caused by *Prevotella oris* (18), and have been reported in both immunocompetent and immunocompromised individuals, typically in the context of periodontal disease (19, 20). In the reported cases of PLA caused by *Fusobacterium nucleatum*, several sources and risk factors have been identified, including Lemierre’s syndrome, long-term denture use, oropharyngeal infections, and diverticulitis of the sigmoid colon leading to bacterial translocation and dissemination. Among these, oral sources are more common, and they often manifest as multiple abscesses (20–22). Past cases have shown that delayed treatment due to misdiagnosis, such as during the COVID-19 pandemic, can lead to severe complications (19). Advanced technologies, such as 16S rRNA gene sequencing, have proven more effective in identifying *Fusobacterium nucleatum* in PLA compared to traditional culture methods (23). *Parvimonas micra* is a Gram-positive anaerobic coccus that can cause bacteremia leading to liver abscesses (24). Liver abscesses caused by *Parvimonas micra* have been reported in two other cases. Both cases involved single microorganism infections, and both were considered to be of dental origin (25, 26). Imaging and blood cultures are crucial for diagnosing the source of infection, and antibiotic treatment is highly effective in resolving the infection (27). Although no cases of liver abscess caused by *Porphyromonas endodontalis* have been reported, it may increase the risk of systemic bacteremia by targeting the GRHL2 and damaging the oral epithelial barrier (28). It is known to be part of the oral microbiome and may lead to microbial infections in the liver (23, 29). Although the anaerobic bacteria identified in this case are more commonly associated with oral infections, our patient did not exhibit any clinical signs of simple or complex periodontitis. Additionally, given her history of chronic colitis and colonic polypectomy, it is hypothesized that the digestive tract is the most likely source of the PLA. However, the exact location remains unknown, as no other definitive source of infection was identified at the time.

How digestive tract anaerobic bacteria invade the liver in this patient is unclear. Pathogenic microorganisms invading the liver primarily cause liver abscess through five main pathways: (1) Biliary pathway, such as various biliary tract diseases and invasive hepatobiliary procedures. (2) Portal vein pathway, often associated with intra-abdominal infections and intestinal infections. (3) Hepatic artery pathway, resulting from the spread of infections originating from other sites. (4) Direct infection, such as penetrating trauma. (5) Cryptogenic infection of unclear origin. Among these pathways, cryptogenic PLA has become the most common mode of infection for PLA in Southeast Asia in recent years. It is generally believed to occur when a primary source of infection elsewhere in the body spreads through the bloodstream to the liver. However, by the time the liver abscess forms, these primary infection sites may have already healed or remain undetected. It has been reported that cryptogenic PLA may be associated with intestinal mucosal damage and could serve as a precursor to colon cancer. Therefore, it’s recommended to consider a colonoscopy examination in cases of cryptogenic PLA. This can help identify some potential underlying colorectal problems or malignancies that may lead to liver abscesses (30). Males, diabetes, and *Klebsiella pneumoniae* infection were more common in cryptogenic PLA and were independent predictive factors than in non-cryptogenic PLA (31). Moreover, polymicrobial infections were less common in cryptogenic PLA. In this report, the patient is a 64-year-old immunocompetent woman who, in the early stages of her illness, did not exhibit any clinical signs related to oral infections. She has no history of diabetes, liver or gallbladder diseases, tumors, immunosuppressive drug use, invasive liver or gallbladder procedures. It’s worth noting that she also has no denture wear and no recent history of dental cleaning. Although she has some chronic colorectal diseases, there are no reports of PLA in the literature. Therefore, this case is an instance of cryptogenic PLA, and it is infected by various digestive tract anaerobic bacteria. By reviewing the currently published case reports, we found that PLA caused by anaerobic bacteria is often associated with cryptogenic infections, which is consistent with our case.

In recent years, high-throughput sequencing technology has rapidly advanced and started to gain popularity in the diagnosis of intra-abdominal abscesses. Compared to traditional methods like microbial culture, mNGS has several advantages in terms of broad-spectrum pathogen detection, shorter turnaround times, and the ability to predict antibiotic resistance through genomic analysis. These advantages of mNGS contribute to improving patient prognosis (32). Zhang et al. evaluated the diagnostic performance of mNGS in detecting intra-abdominal abscesses in the emergency department. They found that mNGS was more sensitive to identify positive cases and pathogen species, providing guidance for clinical management and significantly reducing turnaround time of patients (33). Our case benefited from the application of mNGS, which rapidly provided the basis for identifying the causative pathogens and establishing an accurate antimicrobial treatment plan. The subsequent culture results further confirmed the accuracy of mNGS.

Targeted antimicrobial therapy remains the primary treatment for liver abscesses. It is necessary to empirically use antimicrobial agents covering anaerobic bacteria in patients with PLA and sepsis. Ming et al. found that the in-hospital mortality rate was significantly lower in the group that received anaerobic coverage antibiotics compared to the group without anaerobic coverage. In their multivariate analysis, after adjusting for age and comorbidities, the use of anaerobic antibiotics reduced the in-hospital mortality rate by 42% (34). This study supports that the important role of anaerobic bacteria may have been underestimated in PLA previously, due to the limitations in sensitivity associated with traditional microbial culture methods.

## 4 Conclusion

In summary, this case report presents a rare instance of PLA and sepsis attributed to a mixed infection involving multiple anaerobic bacteria in an immunocompetent patient. Early and accurate detection of causative pathogens was facilitated by the integration of conventional diagnostic methods with mNGS. This case underscores the necessity for heightened awareness of PLA caused by anaerobic bacteria, which may exhibit clinical features akin to those of liver abscesses resulting from other common pathogens. Early use of mNGS in such cases could expedite etiological diagnosis and enable the implementation of targeted antimicrobial therapy, thereby improving patient outcomes. Further studies with larger cohorts are warranted to substantiate these observations and to explore the broader implications for clinical practice.

## Data Availability

The original contributions presented in this study are included in this article/supplementary material, further inquiries can be directed to the corresponding author.
